# VPS54 and the wobbler mouse

**DOI:** 10.3389/fnins.2015.00381

**Published:** 2015-10-21

**Authors:** Thomas Schmitt-John

**Affiliations:** ^1^Neurogenetics, Department of Molecular Biology and Genetics, Aarhus UniversityAarhus, Denmark; ^2^Tauros-DiagnostikBielefeld, Germany

**Keywords:** wobbler, GARP, Vps54, Golgi, vesicle transport, ALS, neurodegeneration

## Abstract

The wobbler mouse is an animal model for human motor neuron disease, such as amyotrophic lateral sclerosis (ALS). The spontaneous, recessive wobbler mutation causes degeneration of upper and lower motor neurons leading to progressive muscle weakness with striking similarities to the ALS pathology. The wobbler mutation is a point mutation affecting Vps54, a component of the Golgi-associated retrograde protein (GARP) complex. The GARP complex is a ubiquitously expressed Golgi-localized vesicle tethering complex, tethering endosome-derived vesicles to the trans Golgi network. The wobbler point mutation leads to a destabilization of the Vps54 protein and thereby the whole GARP complex. This effectuates impairments of the retrograde vesicle transport, mis-sorting of Golgi- and endosome localized proteins and on the long run defects in Golgi morphology and function. It is currently largely unknown how the destabilization of the GARP complex interferes with the pathological hallmarks, reported for the wobbler motor neuron degeneration, like neurofilament aggregation, axonal transport defects, hyperexcitability, mitochondrial dysfunction, and how these finally lead to motor neuron death. However, the impairments of the retrograde vesicle transport and the Golgi-function appear to be critical phenomena in the molecular pathology of the wobbler motor neuron disease.

## Introduction

**Figure 1:**
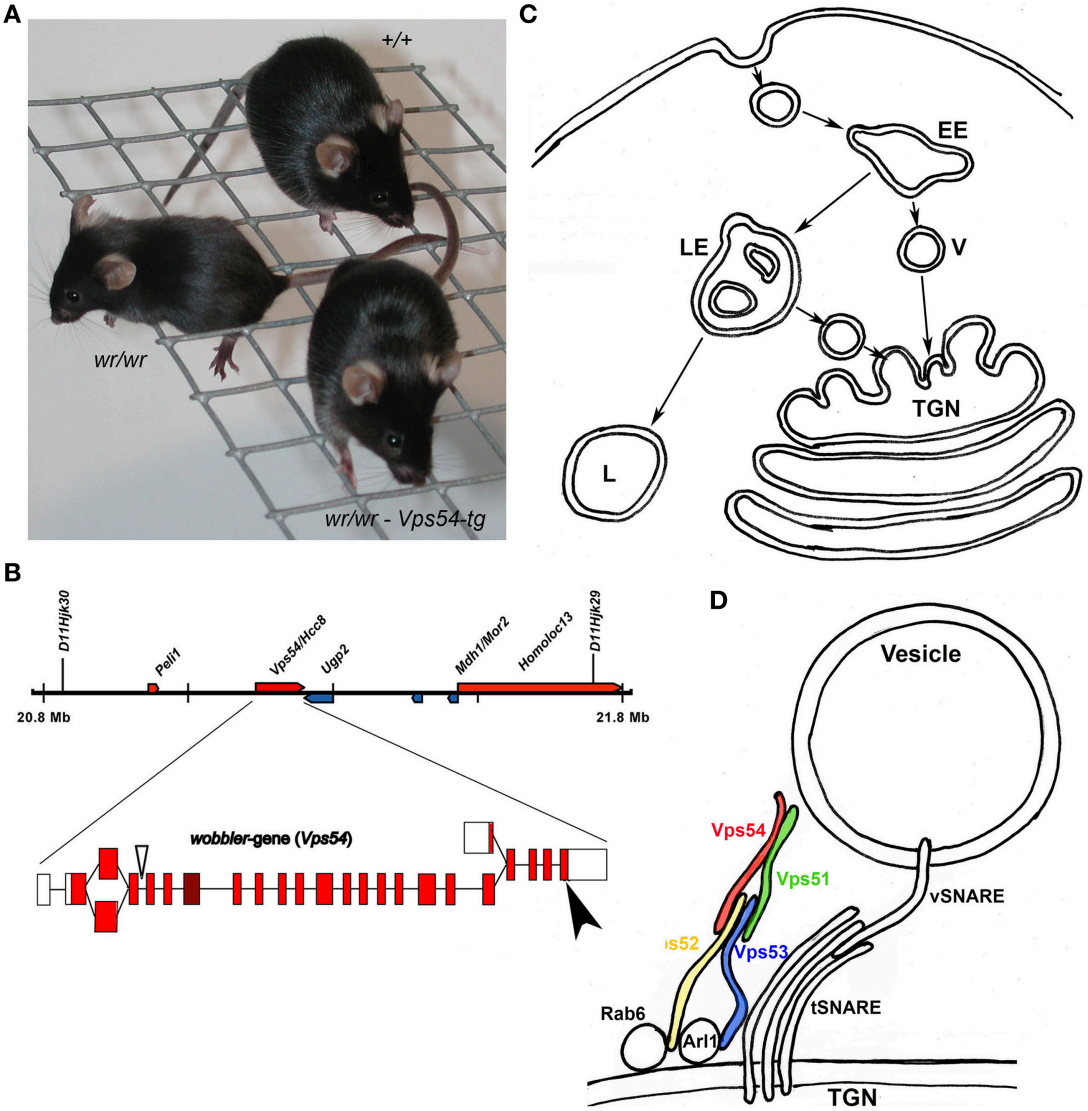
**The wobbler loss-of-function mutation of Vps54**. The figure summarizes the primary defects of the wobbler mutation. **(A)** A Wildtype mouse (+/+), a homozygous wobbler (wr/wr) mouse with defects in grid walking due to muscle weakness, predominantly affecting the fore limbs and a Vps54 transgenic wobbler mouse (*wr*/*wr*-Vps54-tg) with wild type appearance (Schmitt-John et al., 2005). **(B)** Vps54 genomic locus on mouse Chr 11, Exon intron structure, the arrow indicates the wobbler point mutation in exon 24. **(C)** The retrograde vesicle transport route from early (EE), and late endosomes (LE); transport vesicles (V) are tethered to the trans Golgi network (TGN); lysosomes (L). **(D)** The GARP complex consists of Vps51, −52, −53, and −54 and tethers endosome-derived vesicles (V) and mediates vSNARE–tSNARE dependent fusion of the vesicle- and target membrane. The GARP complex interferes thereby with GTP effector proteins Rab6 and Arl1. However, the exact docking site to the vesicle membrane is still unknown. The wobbler point mutation destabilizes the GARP complex and thus, the wobbler mutation causes impairments of the retrograde vesicle transport, but it is still unclear how these impairments induce motor neuron death.

Vps54 was connected with the wobbler motor neuron degeneration by positional cloning (Schmitt-John et al., 2005; Figures 1A,B). Vps54 (vacuolar protein sorting 54) was first identified in yeast in the course of a screening for mutants with mis-sorting of vacuolar proteins (Conboy and Cyert, 2000; Conibear and Stevens, 2000). Vps54 was found to be a component of the Golgi-associated retrograde protein (GARP) complex (Conibear and Stevens, 2000; Figure 1C) and a mammalian homolog was reported (Liewen et al., 2005). The GARP complex belongs to the CATCHR (complexes associated with tethering containing helical rods) group of multi subunit tethering complexes (MTCs) like Dsl1-, COG- and Exocyst complexes (Bonifacino and Hierro, 2011) and specifically tethers endosome derived vesicles to the trans Golgi network. The GARP complex interferes with Rab6 protein and supports the vSNARE–tSNARE dependent fusion of the incoming transport vesicles with the trans Golgi membrane (Figure 1D) and thus has a function in the retrograde vesicle transport. The identification of the mutation causing the motor neuron degeneration of the wobbler mouse in Vps54 suggested a critical role for Vps54, the GARP complex and the retrograde vesicle transport for motor neuron survival (Schmitt-John et al., 2005; Moser et al., 2013).

## Vps54 and the wobbler phenotype

The wobbler mouse was first described by Falconer (1956) and has recently been reviewed (Moser et al., 2013; Ott et al., 2015) as animal model for amyotrophic lateral sclerosis (ALS; Figure 1A). The spontaneous, recessive wobbler mutation (*wr*) was mapped to the proximal mouse chromosome 11 (Kaupmann et al., 1992) and the critical region was refined to a region homologous to human chromosome 2p13 (Korthaus et al., 1997; Resch et al., 1998; Fuchs et al., 2002). By positional cloning the wobbler mutation was identified as a point mutation in the last exon of Vps54 leading to a single amino acid exchange (Schmitt-John et al., 2005; Figure 1B). A successful transgenic rescue approach proved that Vps54 is the wobbler gene (Schmitt-John et al., 2005). A Vps54 null-mutation caused embryonic lethality around day 10.5 of the embryonic development, indicating that the wobbler point mutation is a partial loss-of function allele of Vps54 (Schmitt-John et al., 2005). However, it was demonstrated that the wobbler point mutation causes a destabilization of Vps54 protein and thereby the whole GARP complex (Pérez-Victoria et al., 2010). This leads to a decreased abundance of GARP tethering complexes in wobbler mutant cells, resulting in impairments of the retrograde vesicle transport (Pérez-Victoria et al., 2010; Karlsson et al., 2013). Since the wobbler phenotype closely resembles the human motor neuron disease ALS, the retrograde vesicle traffic is to be considered critical for motor neuron degeneration.

Even though no ALS cases with mutations in VPS54 have been identified yet (Meisler et al., 2008), there is growing evidence that the vesicle transport plays a critical role in human ALS. A subset of familial ALS forms have been attributed to proteins involved in vesicle trafficking, such as Alsin (ALS2; Yang et al., 2001), VABP (ALS8; Nishimura et al., 2004), and CHMP2B (ALS17; Parkinson et al., 2006). Furthermore, Palmisano et al. (2011) could demonstrate accumulation of APP (amyloid precursor protein) in enlarged endosomes in degenerating motor neurons of both wobbler mice and ALS patients, suggesting similar impairments of the vesicle transport.

In case of wobbler mice the primary cause of motor neuron degeneration is the point mutation of Vps54 (Schmitt-John et al., 2005) leading to a destabilization of the GARP complex (Pérez-Victoria et al., 2010) and impairment of the retrograde vesicle transport (Karlsson et al., 2013), which results in protein mis-sorting and accumulation in enlarged endosomal structures (Palmisano et al., 2011). Despite these facts, it is not that simple to explain how the destabilization of a ubiquitously expressed vesicle tethering factor finally leads to the death of motor neurons.

The wobbler motor neuron degeneration shares a number of pathologic features with human ALS like muscle atrophy, astrogliosis, microgliosis (Duchen and Strich, 1968), hyperexcitability (Nieto-Gonzalez et al., 2011), mitochondrial dysfunction (Santoro et al., 2004), axonal transport defects (Mitsumoto et al., 1990), neurofilament aggregations (Pernas-Alonso et al., 2001), and ubiquitin-positive protein aggregations (Dennis and Citron, 2009), as summarized by Moser et al. (2013). All these cellular effects are directly or indirectly connected to the primary cause, the GARP dysfunction, and contribute more or less to the progression of motor neuron death. It is always difficult to distinguish cause from effect or contributing—from concomitant phenomena. However, all mentioned cellular effects are surely interconnected in a complex manner and the GARP complex and Golgi apparatus appear to play a central role in the wobbler motor neuron pathology.

## Vps54 and the GARP complex

Vps54 interacts with Vps51, −52, and −53 in a 1:1:1:1 ratio and forms the GARP complex (Conboy and Cyert, 2000; Conibear and Stevens, 2000) and appears to be predominantly Golgi-localized, tethering retrograde vesicles derived from early and late endosomes (Quenneville et al., 2006). Vps52, −53, and −54 appear to form a core complex, while Vps51 is loosely associated and links the complex to the SNARE Tlg1p (Siniossoglou and Pelham, 2002). Loss-of-function mutations of either of the core components lead to the same phenotype in yeast, decreased growth rate and mis-sorting of vacuolar proteins (Conibear and Stevens, 2000). Double and triple mutants show the same phenotype, while Vps51 mutants display a milder phenotype (Conibear and Stevens, 2000), indicating that the GARP function depends on the function of each of the core components and to a lesser extent on Vps51 (Conibear et al., 2003). In mammals the GARP complex has a similar structure and function (Pérez-Victoria and Bonifacino, 2009; Bonifacino and Hierro, 2011).

Knock down experiments of GARP components in mammalian cell culture (Pérez-Victoria et al., 2008; Pérez-Victoria and Bonifacino, 2009) and the analysis of wobbler mutant cells (Karlsson et al., 2013) clearly indicate that the mammalian GARP complex has an important role for the retrograde vesicle transport and the sorting of proteins, using this transport route as well as sorting receptors like mannose-6-phosphate receptors (Pérez-Victoria et al., 2008). Recent loss-of-function analysis of the GARP complex in HEK293 cells indicated not only retrograde transport defects but also impairments of the anterograde post-Golgi transport of GPI-anchored and transmembrane proteins (Hirata et al., 2015). GARP dysfunction appears to have an impact on both the retrograde and the anterograde vesicle transport and might affect the integrity and function of the whole Golgi apparatus. Complete loss-of-function mutations of murine GARP components cause embryonic lethality. Homozygous deletion of either Vps54 or Vps53 leads to embryonic lethality around day 10.5 of the embryonic development (Schmitt-John et al., 2005; Karlsson et al., 2013), while the null-mutation of Vps52 however, causes even earlier lethality during gastrulation (Sugimoto et al., 2012). This might suggest an additional function of Vps52 in mammals, independent from the GARP complex. Recently, it was reported that Vps51, −52, and −53, but not Vps54, assemble with syndetin to form the EARP complex, which appears to be localized on Rab4-positive recycling endosomes (Schindler et al., 2015). Syndetin appears to fulfil a Vps54-like function in the endosome-associated protein (EARP) complex and binds preferentially to Vps53 (Schindler et al., 2015). Syndetin and Vps54 are probably responsible for the interaction with a specific Rab protein, Rab4 or Rab6 and thereby directing the complex to a specific localization. Heterozygous mutations in Vps53 have been associated with pontocerebellar hypoplasia type 2E (PCH2E) and thus with mental retardation, microcephaly, spastic quadriplegia, and early onset seizures (Feinstein et al., 2014). This finding suggests impairments of both GARP and EARP function due to Vps53 haploinsufficiency. For both PCH2E and the wobbler motor neuron disease swollen vesicles are reported, but no evidence for lysosomal abnormality or lysosomal storage problems was seen (Palmisano et al., 2011; Feinstein et al., 2014).

## Golgi pathology and motor neuron disease

**Figure 2:**
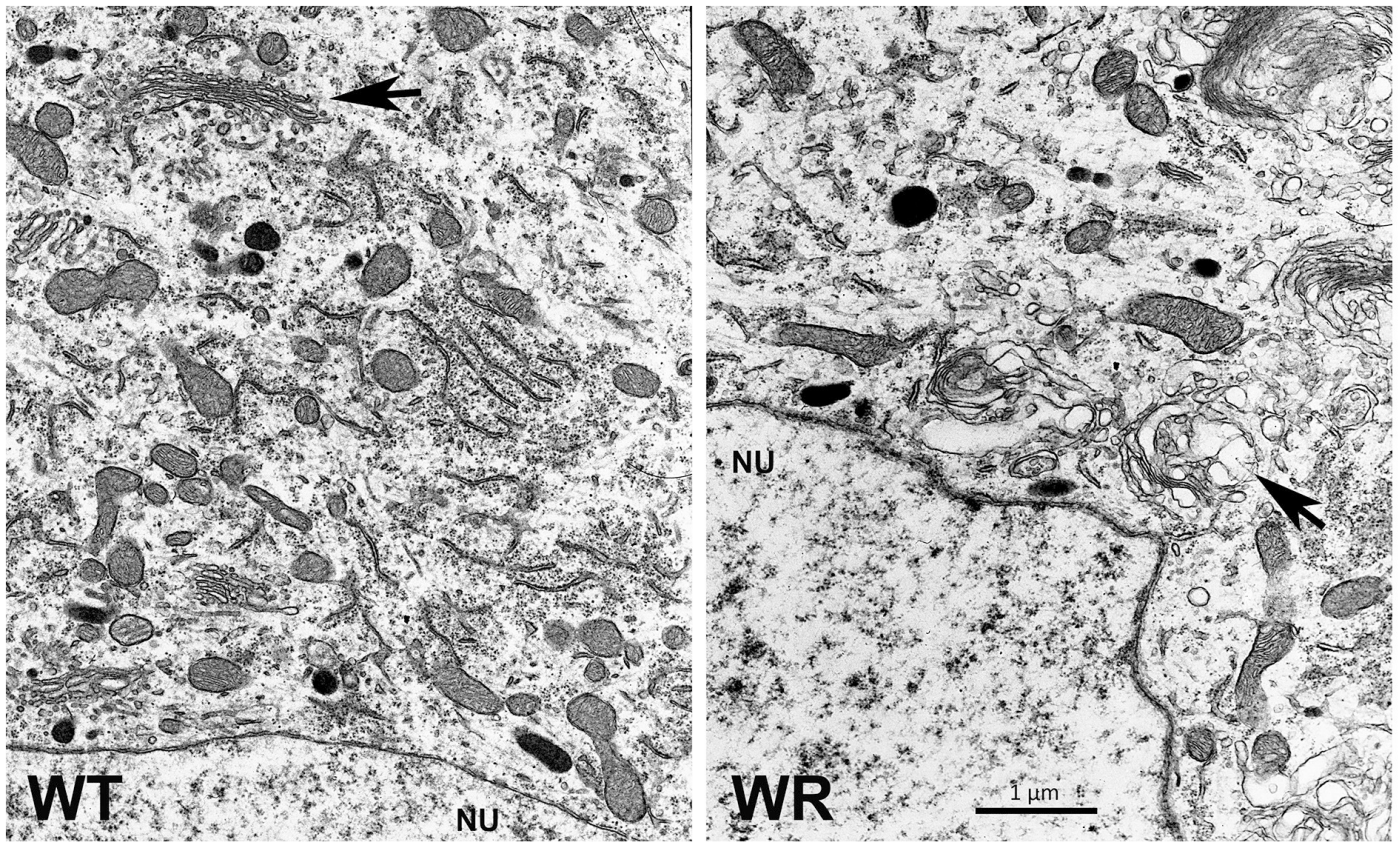
**Electron micrograph of spinal motor neurons**. Regions near the nucleus (NU) of motor neurons from the cervical spinal cords of wild type (WT) and a symptomatic wobbler mouse (WR) is shown. The normal WT- and morphologically affected WR Golgi are indicated by arrows. The image is a gift from Dr. Peter Heimann and shows swollen areas of the WR Golgi, similar to those published by Palmisano et al. (2011).

Golgi function and integrity might have a fundamental significance for neuron function and survival. However, GARP mutant mammalian cells in cell culture show no obvious morphological indications for Golgi-dysfunction or disintegration (Karlsson et al., 2013). In degenerating wobbler motor neurons we observed on electron micrographs enlarged vesicles derived from the Golgi apparatus (Figure 2) in early stages of degeneration and massive vacuolization and fragmentation of the Golgi in late stages (Palmisano et al., 2011). Andrews et al. observed electron dense granular material in close proximity to the Golgi in degenerating wobbler motor neurons (Andrews et al., 1974). In final stages the wobbler characteristic vacuolization is very prominent and the vesicular structures appear to originate not only from the Golgi, but also from the ER (Palmisano et al., 2011). The increased blebbing-out of enlarged vesicles from the Golgi or alternatively the abnormal fusion of incoming vesicles in close proximity to the Golgi argue for a Golgi-dysfunction and in later stages a Golgi fragmentation, which might be a hallmark of the wobbler motor neuron degeneration. Whether the enlarged Rab7-positive, APP containing structures, described by Palmisano et al. (2011), are identical with the vacuoles with low electron density (Andrews et al., 1974; Palmisano et al., 2011) is not yet proven but likely, since these structures are found in the same subcellular location and size range.

Golgi fragmentation has been shown for ALS patients (Stieber et al., 1998) and SOD1 G93A transgenic fALS animal models (Mourelatos et al., 1996) by immunostaining for MG160, a Golgi-resident marker protein. It could also been shown that ALS neurons with TDP-43 aggregates also display Golgi-fragmentation (Fujita et al., 2008). For wobbler mice Golgi-fragmentation has not yet been investigated by anti MG160 immunostaining, but the evidence from electron microscopy (Andrews et al., 1974; Palmisano et al., 2011) and the observation of cytoplasmic TDP-43 aggregations in degenerating wobbler motor neurons (Dennis and Citron, 2009), makes it likely that the Golgi-fragmentation in wobbler mice resembles that found in ALS patients and SOD1 transgenic mice.

Taken together, wobbler mice develop motor neuron degeneration, which closely resembles human ALS and the primary cause is a partial loss-of-function mutation of Vps54. The wobbler point mutation destabilizes Vps54 protein and thereby the GARP vesicle tethering complex leading to impairments of the retrograde vesicle transport. While most cell types can cope with these impairments, motor neurons develop a number of pathological phenomena. In motor neurons the retrograde vesicle transport defects lead to enlarged endosomal structures (Palmisano et al., 2011), Golgi-dysfunction, impairments of the anterograde and retrograde axonal transport (Mitsumoto et al., 1990), TDP-43- and ubiquitin positive protein aggregations (Dennis and Citron, 2009), neurofilament aggregations (Pernas-Alonso et al., 1996), mitochondrial dysfunction (Santoro et al., 2004), and hyperexcitability (Nieto-Gonzalez et al., 2011). In the final stage motor neurons display a pronounced vacuolation (Duchen and Strich, 1968; Palmisano et al., 2011). The wobbler motor neuron degeneration is even in early stages associated with pronounced astrogliosis and microgliosis (Laage et al., 1988; Rathke-Hartlieb et al., 1999), which might exacerbate the neurodegeneration. However, it is still unclear how the various phenomena cohere or interdepend and which role the Golgi-dysfunction plays in wobbler motor neuron disease.

### Conflict of interest statement

The author declares that the research was conducted in the absence of any commercial or financial relationships that could be construed as a potential conflict of interest.
